# MPLA Case 4: A physicist’s consult with a patient

**DOI:** 10.1002/acm2.13211

**Published:** 2021-04-03

**Authors:** Lee Xu, Leonard Kim, Patricia Sansourekidou, Dongxu Wang

**Affiliations:** ^1^ Department of Radiation Oncology University of Pennsylvania Philadelphia PA USA; ^2^ Department of Radiation Oncology MD Anderson Cancer Center at Cooper Camden NJ USA; ^3^ Department of Radiation Oncology University of New Mexico Comprehensive Cancer Center Albuquerque NM USA; ^4^ Department of Medical Physics Memorial Sloan Kettering Cancer Center Middletown NJ USA

**Keywords:** case study, leadership, MPLA, professionalism

*This work of fiction is part of a case series by the Medical Physics Leadership Academy (MPLA) demonstrating the use of case studies as an educational method for the development of professionalism, leadership, and communication skills within the medical physics community* ^1^

*A facilitator's or self‐study guide is available. Please contact the MPLA Cases Subcommittee (* https://www.aapm.org/org/structure/default.asp?committee_code=MPLACA *) for access*.

## 1 Synopsis

It was an unusual day for Wendi Lin, a physicist who was just 10 months into her clinical career and very much still getting the hang of things. At the request of Dr. Abbasi, one of the radiation oncologists, Wendi was preparing to enter a consult room to explain to a patient — who was adamant on receiving proton therapy — the practical differences between two particles used in treatment. Although initially surprised by Dr. Abbasi’s request, Wendi was rather excited to meet with the patient, for she had always enjoyed interacting with patients in the past. This time, however, the encounter did not go as she had expected.

## 2 The Request

“Wendi?” Dr. Abbasi said, knocking on her office door. “It’s John.”

“Hi John, please come in,” Wendi responded, looking up from her computer. “How are you doing?”

“Good, good,” Dr. Abbasi replied as he entered the room. “Do you have a moment by chance?”

“Yeah of course, what’s going on?”

Dr. Abbasi sat down, tucking the end of his white coat neatly beneath him. He crossed one leg over the other and rested his hands on his knee. “Listen,” he said, letting out a sigh. “I have a little situation I was hoping you could help me with.”

“Sure,” Wendi answered, intrigued. Over the past few months, Wendi had gotten along with Dr. Abbasi quite well, but had never encountered him like this. “What can I do for you?”

“I just came from the clinic. I have a patient—Vaughn—who was supposed to start VMAT five weeks ago. It’s a prostate. The problem is, he refuses to be treated with photons. He only wants protons.”

“I see…” Wendi replied, wondering how she could possibly help.

“I agreed to switch him over a month ago, but we still haven’t heard back from his insurance. It could be a few more weeks,” he continued. Dr. Abbasi took a deep breath in, taking a moment to organize his thoughts. “I just tried to convince him again to start treatment, but he just won’t budge. He is convinced that proton therapy is some kind of miracle therapy.”

“Hmm, that does sound like quite the situation…” Wendi responded, hoping to lighten the mood. “And why would he think that?”

“After our initial consult he did some research and concluded that protons are the way to go. I tried explaining to him that there are pros and cons to each method, but he just doesn’t seem to buy it.” Dr. Abbasi took a moment to organize his thoughts. “Do I think he’ll tolerate protons better? Maybe. But the point is his outcome is going to be similar either way…as long as he starts treatment that is. His PSA is rising.”

“Oh, well it definitely sounds like he’s got his mind made up,” Wendi replied, somewhat impressed. She paused to see if Dr. Abbasi would prompt her. “So what can I do to help?”

“The patient is actually still here, right now,” Dr. Abbasi responded, uncrossing his legs. “I was wondering if you would be willing to talk to him.” “Just briefly,” he added. “Maybe you could clarify some of his notions on photons vs protons from a physics perspective?”

“Oh really?” Wendi responded. “I mean sure, I wouldn’t mind doing that,” she added.

“Great, I’d really appreciate it,” Dr. Abbasi said as he stood up. “He should still be in the clinic…you can just ask one of the nurses which room he’s in. It’s Richard Vaughn: V‐A‐U‐G‐H‐N.” Dr. Abbasi turned to leave the room before peering back, his phone now buzzing in his hand. “Thanks again Wendi.”

## 3 Wendi’s preparation

Wendi was taken aback. Out of all the scenarios she had imagined happening, this was not one of them. Physicists were not supposed to consult with patients, were they? Even if they were, surely no one had told her or prepared her for it. But as much as her anxiety ramped up, surprisingly so did her excitement. After all, she knew how to talk to patients from her days as a hospital volunteer in high school. In fact, she enjoyed doing it, so what was there to worry about? Wendi reminded herself why she became a medical physicist in the first place — it was for moments like these. Ever since college she wanted to work in healthcare and help patients get the treatments they needed. Her training in physics provided her knowledge on the intricate behavioral differences between various particles. Now the question was whether she knew how to convey those differences to a patient.

Wendi opened Mr. Vaughn’s electronic chart and pulled up both the VMAT (photon) and PBS (proton) plans in the treatment planning system. She noticed that both plans exhibited good PTV coverage and dose conformality. The VMAT plan utilized two full arcs whereas the PBS plan utilized two lateral fields. The dose distribution in the VMAT plan was fairly homogeneous with the low dose evenly distributed along the rotational axis of the beam. Compared to the VMAT plan, the PBS plan had a lower mean rectal and bladder dose, but the entrance dose was higher. The PBS plan also had a lower integral dose, as expected. Overall, Wendi found both plans to be reasonable, but the proton plan did have a more favorable dose distribution. Wendi wondered what she would say if the patient asked about this. Would knowing this information do more harm than good? She decided she would not bring it up of her own volition.

Wendi put on the white coat hanging behind the door, smoothing out the wrinkles as best as she could. As she made her way toward the clinic, a flood of thoughts rushed through her head. Ever since she had started, most of the work she had done was behind the scenes. She reviewed patient charts in her office, resolved treatment issues from the console areas, and waited patiently for the clinical days to end before hopping on the machines after hours. Aside from the select few on the brachytherapy team, the physicists almost never interacted with any of the patients. She wondered whether they even knew she existed. It is not like medical physicists were the physicists typically portrayed on T.V. or in the movies. Wendi glanced at the time on her phone and picked up her pace — she had lost track of time. It had already been 20 minutes and she did not want the patient to wait any longer.

## 4 Wendi’s Consult

“Hi, Mr. Vaughn? I’m Wendi—I’m one of the physicists here,” she said in a cheerful tone. Wendi reached forward and shook his hand. “It’s nice to meet you.”

“Very nice to meet you Wendi. I have heard that medical physicists are employed here to oversee radiation technology.” he replied. Mr. Vaughn was a gentleman in his late 60’s, dressed in a brown tweed sport coat with matching slacks. A check‐patterned flat cap adorned his head.

“Sorry to keep you waiting—I heard from Dr. Abbasi that you might have some questions regarding photon and proton therapy?”

“Not really any questions, but I don’t want photon therapy. When will the insurance be approved for protons?”

“Well,” Wendi sighed. “That’s something I could check with billing for you, but usually it takes anywhere from four to eight weeks. In the meantime, however, Dr. Abbasi would like you to start treatment with photons. What are your hesitations regarding that?”

“It seems proton therapy is the newest technology—I’ve spent some time reading about it. I would like the best treatment available.” He responded.

“Yes of course—I totally understand. The interesting thing about your case is that proton therapy isn’t necessarily better than photon therapy. With proton therapy there is a theoretical benefit in normal tissue sparing, but there really hasn’t been anything definitive to show this. Like you mentioned, it is a newer technology, and because of that it also comes with uncertainties that haven’t been studied as well.” Wendi explained.

Mr. Vaughn sat up in his chair, taking a moment to adjust his posture. His demeanor seemed to change.

“What kind of uncertainties are you talking about?” he asked. “I thought protons could be directly targeted to the tumor.”

“Well, yes ideally we want the proton beam stop in the tumor itself, but protons suffer from something known as end‐of‐range uncertainty. And this is a phenomenon where the end of the beam becomes somewhat blurred out. Because of this, we don’t always know precisely where the proton beam ends and have to add a margin to account for it.”

“What do you mean you don’t know where the proton beam ends?” Mr. Vaughn said, somewhat agitated. “I thought you guys were the experts at this.” Wendi could sense she had said something she shouldn’t have.

“I mean… we do know where the proton beam ends,” Wendi countered, somewhat flustered. “It’s just that protons are very sensitive to tissue density which affects how far they travel in a medium. And this is especially important in the pelvic region where you have bone, fat and muscle. Of course, we always try our best to account for this.”

“Is that only for protons then, or should I be worried about photons too?” Mr. Vaughn asked.

“Photons are affected too, to an extent,” Wendi answered. “But protons are especially affected just based on their nature—being charged—and how they interact with matter. Protons are also plagued by uncertainties in their biological effect, but that’s an entirely separate topic of its own.”

“So you guys also don’t know the biological effect of protons? What else don’t you know? Should I be coming here at all?” Mr. Vaughn asked, raising his voice. Wendi could tell she was quickly losing control of the conversation.

“Mr. Vaughn,” Wendi responded, somewhat distressed. “Proton therapy really is an excellent modality, but it’s certainly not perfect. Are there cases where protons are the preferred way to go? Definitely. But then there are cases like yours, where at the moment the benefit isn’t as clear. Photon therapy has its own downsides but it’s been around for quite a while now and is a tried and true method. I’m sure Dr. Abbasi reviewed your case extensively before deciding on the appropriate treatment for you.”

Mr. Vaughn had somewhat of a blank stare on his face as if he were processing his thoughts. Wendi wondered if she had given him too much information at once. He shifted his posture again before letting out a small sigh.

“We all have your best interests here I can promise you that,” Wendi reassured him. “Why don’t you take a day or two to think about it and get back to us. Does that sound alright?”

“Fine,” Mr. Vaughn muttered. “I’ll need some time to give it another thought. It’s just a lot to take in.”

“I completely understand,” Wendi said.

Wendi closed the door behind her, feeling a mixed wave of relief and unease rush over her. She completed her first ever patient consult as a physicist, even if it did not go as well as she hoped. At least the patient was somewhat skeptical of proton therapy now, she joked to herself. On her way out of the clinic, Wendi wondered if she would ever return to do another consult. She definitely wanted to work on her communication skills beforehand if so. Until then, she would send Dr. Abbasi a quick update before heading back to her office to continue on with her weekly chart checks.

## Conflict of Interest

The authors report no conflicts of interest and are solely responsible for the content and writing of the paper.
